# Editorial: Emerging and Re-emerging Organizational Features, Work Transitions, and Occupational Risk Factors: The Good, the Bad, the Right. An Interdisciplinary Perspective

**DOI:** 10.3389/fpsyg.2018.01533

**Published:** 2018-08-23

**Authors:** Giulio Arcangeli, Gabriele Giorgi, Nicola Mucci, Jean-Luc Bernaud, Annamaria Di Fabio

**Affiliations:** ^1^Department of Experimental and Clinical Medicine, University of Florence, Florence, Italy; ^2^Department of Human Sciences, European University, Rome, Italy; ^3^Conservatoire National des Arts et Métiers, Paris, France; ^4^Department of Educational Sciences and Psychology, University of Florence, Florence, Italy

**Keywords:** organizational psychology, organizational risks, work-related stress, innovation, engagement, welfare, organizational justice, occupational medicine

## Introduction

This special issue aims to provide an overview of the profound changes that have interested the labor market in the last decade all over the world. These rapid and profound transformations have, on the one hand, generated new opportunities for both employers and employees, but, on the other hand, they have led to the re-actualization of old organizational risks as well as the emergence of new occupational risks.

In such a context, the stakeholders had to suddenly face a new context of challenges and critical issues in the workplace and, therefore, it is not surprising that scientific research is increasingly focusing on perceived organizational support, commitment in organizational context, socialization processes, changes in capacity of organizations, perceived organizational justice, occupational ergonomics, and motivation.

The European Agency for Safety and Health at Work (EU-OSHA, 2016) has identified three key points that describe the ongoing evolution in the modern labor market: globalization, technical innovation, and aging of the population. First, some potential occupational risks, for many years considered old, are reappearing in organizations: intensive fear and worries, organizational anxiety, boredom, physical violence, alienation, segregation, loneliness, and isolation. Moreover, the perceived re-emerging organizational characteristics seem to be of utmost importance for companies.

The 33 manuscripts selected for this special issue are mainly empirical contributions, written by authors belonging to different disciplinary sectors and coming from different geographical regions. They therefore offer different perspectives on labor transitions and occupational risk factors, also contributing to promoting an interdisciplinary and international approach to research that will allow the progress of science in the field of occupational health and safety (H&S).

The manuscripts, when considered together, bring out three important aspects. First of all, the issues concerning the re-emergence and the emergence of occupational risks and professional opportunities are particularly current in the light of the continuous changes and of the instability of companies in the global economy. Secondly, employee health and well-being are crucial in a time of current global financial crisis and economic pressures on companies. Finally—considering the huge social, political and environmental implications—in this special issue is clearly emerged the importance of evaluation and prevention of psychosocial risks and work-related stress.

## Overview of articles in this research topic

The 33 papers published in the special issue addressed these issues in a variety of ways. There are: 23 original research; 6 perspectives; 3 conceptual analysis; and one review.

Following the setting of this Research Topic, the articles can be divided into two macro-categories: one “positive” (*the good, the right*) and the other “negative” (*the bad*). The first macro-category emphasizes the importance of health promotion and of the elements oriented to improve health and well-being of workers. The second macro-category places greater emphasis on the risk factors that the organization has to properly assess for the purpose of intervention in order to preserve health and safety of workers.

Furthermore, this Research Topic was focused on emerging and re-emerging organizational features in the contemporary occupational world. The received contributions highlight the strong need of studying the emerging phenomena both for health and for wellbeing of workers. However, it is equally important that the research also takes into account the “old” constructs that may acquire different and changing meanings in today's occupational landscape, by virtue of the temporal and spatial context.

Thirteen manuscripts (6 original articles; 4 perspectives; 3 conceptual analysis) focus on the “positive” aspects (*the good, the right*) of work organizations, and highlight, at the same time, mainly emerging issues (well-being, performance, personal and organizational growth, healthy business, sustainable development, Industry 4.0, gratitude, etc.).

Mariani et al. consider safety climate in a warehouse and wants to analyze the Leader–Member Exchange (LMX) role in respect to safety performance. Survey data were collected from a sample of 133 full-time employees in an Italian warehouse. The study shows that the different aspects of leadership processes interact in explaining individual proficiency in safety practices.

Vignoli et al. using the Job Demands-Resources model, demonstrate how a mixed methods approach to conducting screening enables the identification of potential context-dependent demands and resources in the workplace, which should to be targeted by the intervention. The study confirms that mixed methods approach is useful in occupational health intervention research and offers a way forward on helping organizations prioritize their intervention activities.

Palazzeschi et al. address innovation in organizations in the scenario of Industry 4.0, including technological innovation and psychological innovation. This perspective article also suggests new directions in a primary prevention perspective for future research and intervention relative to innovation and innovative work behaviors in the organizational context.

Di Fabio et al. examine, in a sample of 258 Italian workers, the relationship between the Intrapreneurial Self-Capital Scale (ISC) and well-being (hedonic well-being and eudaimonic well-being) controlling for the effects of personality traits, administrating the Big Five Questionnaire (BFQ), the Intrapreneurial Self-Capital Scale (ISCS), the Satisfaction With Life Scale (SWLS), and the Flourishing Scale (FS). Hierarchical regression analyses showed that ISC explained a percentage of incremental variance beyond that explained by personality traits in relation to both life satisfaction and flourishing.

Ariza-Montes et al. assess the link between authenticity and subjective wellbeing within the rarely explored context of faith-driven organizations, where the management of emotions attains a particular significance. Specifically, this study links authenticity with subjective wellbeing among the distinct groups that shape a large international Catholic organization. Relatedly, the majority of studies featured in this special issue explored different organizational features linked to labor market changing.

Petrovic et al. analyze the psychometric properties of the Serbian versions of the UWES-17 and UWES-9. The sample consisted of 860 employees from a number of organizations and jobs across Serbia. The study contributes to enhanced understanding of work engagement by offering an insight from the Serbian cultural and economic context, significantly different from the UWES originating setting.

Di Fabio et al. in a perspective article, reviews the construct of gratitude. In organizations, gratitude is now thought to be crucial to employees' efficiency, success, and productivity while also improving organizational citizenship behaviors, prosocial organizational behavior, and the organizational climate.

Another perspective article by Di Fabio deals with the concept of healthy organizations and starts with a definition of healthy organizations and healthy business. The focus is not on deficiency and failure but on a positive organizational attitude that proposes interventions at different levels: individual, group, organization, and inter-organization. Healthy organizations need to find the right balance between their particular situation, sector, and culture, highlighting the importance of well-being, and sustainability.

A third perspective article by Di Fabio discusses the contribution of the psychology of sustainability and sustainable development to well-being in organizations from a primary prevention perspective. It deals with sustainability not only in terms of the ecological, economic, and social environment but also in terms of improving the quality of life of every human being. The psychology of sustainability and sustainable development is seen as a primary prevention perspective that can foster well-being in organizations at all the different levels going from the worker, to the group, to the organization, and also to inter-organizational processes.

Dell'Aversana and Bruno, in a perspective article, explore the perceptions of healthcare providers in managing diversity and the strategies used to meet health needs at a professional and organizational level. Findings indicated that dealing with diversity poses challenges for healthcare providers, by confronting them with multilevel barriers to quality of care.

A conceptual analysis by Herrera-Sanchez et al. focuses on the implementation process and attempts to identifying the main factors that contribute toward ensuring a greater success of occupational health and safety interventions conducted at the organizational level. They also propose some steps that can guide a successful implementation.

A conceptual analysis by Theeboom et al. describe which competencies of coaches are crucial in the different stages of change that coaching aims to bring about. The framework delineated in this paper contributes to the understanding of coaching as a tool to assist employees in dealing with the challenges of an increasingly dynamic work-environment and yields concrete suggestions for future theory development and research on coaching.

Graffigna discuss the results of a conceptual analysis of the literature conducted in order to investigate overlapping features and areas of divergence among three different areas of investigation and application of the engagement phenomenon in organized settings: the domains of employee engagement, consumer engagement, and patient engagement.

Nine manuscripts (8 original articles and a perspective) are focused on the “positive” aspects (*the good, the right*) of work organizations, and highlight, at the same time, mainly re-emerging issues (organizational justice, job demands, organizational constraints, role ambiguity, inclusive leadership human performance, etc.).

Lee et al. explore the linking mechanisms and conditional processes underlying the relationship between psychological voice climate and individual change readiness in a sample of 187 full-time employees.

Qi and Liu investigated the impact of inclusive leadership on employee voice behavior and team performance through caring ethical climate evaluating the model with a time-lagged data of 329 team members from 105 teams in six cities in China. This study revealed the mechanism of the positive cross-level effects of inclusive leadership on the caring ethical climate, employee voice behavior, and team performance.

Bar-On describe the development as well as the initial norming and validation of the Multifactor Measure of Performance™ (MMP™), which is a psychometric instrument that is designed to study, assess and enhance key predictors of human performance to help individuals perform at a higher level.

Loscalzo et al. deep the analysis of the wellbeing of peacekeepers military. They founded that peacekeepers have higher levels of psychological resources (i.e., self-efficacy, self-esteem, social support) and quality of life (i.e., higher life satisfaction and lower general stress).

The aim of Abessolo et al. study was to use Schwartz's model of structural values to empirically explore the relationships and structural correspondences among basic values, career orientations, and career anchors in an heterogeneous sample of 238 employees from French-speaking Switzerland. The results showed that it was possible to meaningfully position both career orientations and career anchors in Schwartz's values structure.

Pan et al. examined the effects of organizational justice (OJ) on positive organizational behavior POB of employees with two different studies, a large-sample survey and a situational experiment. In the first study, a total of 2,566 employees from 45 manufacturing enterprises completed paper-and-pencil questionnaires assessing OJ and POB of employees. In the second study, 747 employees were randomly sampled to participate in the situational experiment with 2 × 2 between-subjects design.

Kowalczuk et al. evaluate the correlations between different aspects of 789 nurses' psychosocial working conditions. The results show that perception of the need for changes was influenced by the assessment of job demands, components of the control scale and, most of all, the scale of social support.

Since atypical forms of employment have substantially increased in the labor market, Kottwitz et al. building on research regarding organizational constraints and role ambiguity, hypothesize that the paucity of information is negatively related to job satisfaction. Multiple regression analyses further revealed interaction effects of paucity of information and form of employment. Specifically, the negative correlation of paucity of information with global as well as satisfaction with the social climate was stronger for employees' holding more than one job.

Bruno et al. conducted a survey among 57 Health Department directors belonging to the National Health Service in the North of Italy in 2016 with the aim to explore how different leaders' behaviors (task-oriented and relationship-oriented) interact with CO of health organizations. Specifically, the aim of the paper was to contribute to this topic, by considering the leaders' point of view.

Three original articles are focused on the “negative” aspects (*the bad*) in the work organizations, and highlight, at the same time, mainly emerging issues (such as age, work ability, future time perspective, and expatriations).

Converso et al. aims at examining the role of job and personal resources between age and work ability within 333 nurses. Multiple linear regression showed that age is significantly and negatively associated to work ability, and that job resources (e.g., decision authority and meaning of work) and personal resources (e.g., hope and resilience) moderate the relationship between age and work ability.

Kerry and Embretson analyzed FTP (Future time perspective) in an experimental manipulation of subjective life expectancy (SLE). Results indicate general support for decreasing age-change in FTP, indicated by independent-sample *t*-tests showing lower FTP in the “Die-by” framing condition.

In the article by Jannesari et al. is examined the role of psychological availability as a means of psychological engagement between self-initiated expatriates (SIEs) and their host-country nationals (HCNs) colleagues during their work and interaction adjustment. The study demonstrated the value of proactive personality as an antecedent effect and supportive supervisor relations as a moderating effect and investigated how these factors can lead to a sense of psychological availability and boost psychological engagement between SIEs and HCNs in order to improve the adjustment between them.

Eight manuscripts (7 original articles and a review) are focused on the “negative” aspects (*the bad*) in the work organizations, and highlight, at the same time, mainly re-emerging issues (such as burnout, job insecurity, long working hours, specific occupational stressors as robberies, etc.).

Trifiletti et al. with the aim of extending the Anxiety Buffer Disruption Theory (ABDT), argue that high levels of burnout may disrupt the anxiety buffer functioning that protects people from death concerns. Participants were 418 nurses, who completed a questionnaire including: a mortality salience (MS) manipulation, a delay manipulation, and measures of burnout, work-related self-efficacy, and representation of oneself as a valuable caregiver.

Golonka et al. refer to cognitive aspects of burnout as the effects of long-term work-related stress. The purpose of the study was to investigate electrophysiological correlates of burnout to explain the mechanisms of the core burnout symptoms: exhaustion and depersonalization/cynicism.

In an EEG study, Golonka et al. focuses on analyzing event-related potentials (ERPs): N170, VPP, EPN, and LPP, as indicators of emotional information processing. The results show that burnout subjects, as compared to the control group, demonstrate significantly weaker response to affect-evoking stimuli, indexed by a decline in VPP amplitude to emotional faces and decreased EPN amplitude in processing emotional scenes.

The study by Soler-Gonzalez et al. assessed the relationship between absence (loneliness) and presence (empathy) of human connections with the occupational well-being of healthcare professionals in a sample of 628 healthcare professionals working in Spanish public healthcare institutions. The findings support an important role for empathy in the prevention of work stress in healthcare professionals. They also confirm that loneliness, as a multidimensional and domain specific experience, is detrimental to occupational well-being.

Wagner-Hartl and Kallus suggest that from a psychophysiological point of view long working hours were more demanding for normal hearing employees. In total, 51 white-collar workers, aged between 24 and 63 years, participated in the laboratory study. The results show no significant effects for age and hearing impairment on the intensity of subjective consequences (perceived recovery and fatigue, subjective emotional well-being and physical symptoms) of long working hours.

Chirumbolo et al. proposed the Job Insecurity Integrated Model aimed to examine the effects of quantitative job insecurity and qualitative job insecurity on their short-term and long-term outcomes. This model was empirically tested in two independent studies, hypothesizing that qualitative job insecurity mediated the effects of quantitative job insecurity on different outcomes, such as work engagement and organizational identification (Study 1), and job satisfaction, commitment, psychological stress, and turnover intention (Study 2).

Setti et al. explore to what extent experiencing robberies and/or thefts at work affect workers' mental health, coping-self-efficacy, social support seeking, workload, and job satisfaction. The results indicated that victims of thefts and robberies experienced greater workload, higher psycho-physical complaints, and greater tendency to seek social support in comparison with their non-affected counterparts.

The only review of this Research Topic, by Giorgi et al. assess, on the MEDLINE® database, the work-related stress in the banking sector. There was uniform agreement among the studies that stress in the credit industry is now at critical levels, and that it can have deleterious psychological effects on workers, and on their physical health, and that organizations, too, are affected. Most studies showed that mental health problems had increased in the banking sector, and that they were stress-related.

## Conclusion

Overall, the papers in this special issue report findings from a cumulative sample of nearly 31,000 workers and perspectives from 100 authors. They suggest that emerging and re-emerging organizational features and occupational risk factors may be enhanced by rapid transformations in both in organizations and in the workers (i.e., numerous changes in demographics, society, working processes, productive rhythms, and environment) and provide several perspectives and instruments to orientate both practice and research in the future.

We believe that the best starting point for making a correct assessment of the future of work is to analyze the current situation of the same. In this context, today there is a strong need to focus both on emerging occupational risks and on the reactivation of occupational risks that are now considered historic.

We would like to highlight a motto of the Business@Health Laboratory of the European University of Rome (www.uerbusinesshealth.com): “*business doesn't exist without workers' health & workers' health is business.”* Our hope is that this special issue will stimulate researchers in many disciplines to broaden their perspectives and horizons with the goal to integrate occupational H&S politics as part of activities oriented to promote at the same time health, well-being, performance, and productivity (e.g., Sagha Zadeh et al., 2018; Sorensen et al., 2018; Di Fabio and Kenny).

In fact, the adoption of multidisciplinary and integrated methodologies in the H&S management in the workplace is emerging. Such an approach is applicable in any employment context, particularly in larger companies. A careful assessment of the organizational and psychosocial risks and a subsequent adoption of truly effective measures can bring—in addition to the H&S benefits of workers—advantages for the companies in terms of increasing productivity and reducing injury risk. In this view, all the policies and the intervention strategies proposed in this special issue can be considered more as an opportunity than as a cost for the employers. This appears as the only road that can now be pursued to guarantee the competitiveness and sustainability of European companies in an increasingly varied and globalized market.

## Author contributions

GA, GG, NM, J-LB, and AD equally contributed to all the following issues of the Editorial: conception of the work; acquisition, analysis, or interpretation of data from the contributions; drafting the work and critically revising it; final approval of the version to be published; agreement to be accountable for all aspects of the work in ensuring that questions related to the accuracy or integrity of any part of the work are appropriately investigated and resolved.

## Conflict of interest statement

The authors declare that the research was conducted in the absence of any commercial or financial relationships that could be construed as a potential conflict of interest.
